# Colorimetric Determination of Peroxides: A New Paper-Based Sensor for Detecting Hexamethylene Triperoxide Diamine (HMTD)

**DOI:** 10.3390/s26030904

**Published:** 2026-01-30

**Authors:** Wiktoria Furmanek, Viktoriia Lastivka, Piotr Kasprzak, Izabela Mazur, Piotr Andrzej Baran, Wawrzyniec Pniewski, Łukasz Kukiełczyński, Mariola Koszytkowska-Stawińska, Ilona Grabowska-Jadach, Michał Chudy, Katarzyna Tokarska, Kamil Żukowski, Artur Dybko

**Affiliations:** 1 Faculty of Chemistry, Warsaw University of Technology, Noakowskiego 3, 00-664 Warsaw, Polandartur.dybko@pw.edu.pl (A.D.); 2 Military Institute of Armament Technology, Wyszynskiego 7, 05-220 Zielonka, Poland; 3 The Centre for Advanced Materials and Technology, Warsaw University of Technology, Poleczki 19, 02-822 Warsaw, Poland

**Keywords:** paper-based sensor, hexamethylene triperoxide diamine detection, explosive materials

## Abstract

Homemade explosives (HMEs) are of increasing interest to security forces worldwide due to their widespread utilization by terrorists. Their synthesis is relatively straightforward, requiring only a few readily available chemical compounds. Among the most popular HMEs are organic peroxides, specifically triacetone triperoxide (TATP) and hexamethylene triperoxide diamine (HMTD). This manuscript reports on a paper-based sensor developed for the detection of HMTD. The sensor facilitates fast, inexpensive, and simple detection of this peroxide. The developed sensor was compared with well-established commercial and in-house-developed iodometric tests typically used for peroxide determination. The colorimetric principle was based on the use of a potassium iodide and citric acid solution applied to a paper substrate. The HMTD and TATP samples were prepared in an acetone–water mixture (1:1, *v*/*v*). The sensor was able to detect HMTD down to a limit of 0.01 mg/mL. The developed sensor does not exhibit cross-reactivity with other explosives, including TATP. Furthermore, an HSV analysis of the photographs was performed using the Trigit application.

## 1. Introduction

Explosives are a diverse group of extremely hazardous substances that, in the wrong hands, become lethal weapons. Homemade explosives (HMEs) represent a particular challenge to public safety, as their ingredients are widely available in various supermarkets, and the synthesis itself is quite simple and does not require the use of complex equipment. The danger posed by HMEs can be seen in the terrorist attacks carried out by the Islamic State in 2015 in Paris [1] or in Brussels in 2016 [2]. Authorities are focusing a major part of their efforts on counter-terrorism and are taking various actions to detect HMEs in order to prevent terrorist attacks. Different locations and circumstances require different detection methods for these hazardous substances. A range of equipment and analytical methods are utilized at airports and public places to detect explosive materials. Among the most popular analytical techniques used to detect HMEs are chromatographic techniques, including both liquid [3] and gas [4] chromatography. It is also very common to perform analyses using Raman spectroscopy [5,6,7]. Since most explosive materials do not exhibit any absorbance changes in the visible or UV range of the spectrum, these well-known spectroscopic techniques are often unsuitable for developing devices for detecting such materials. Examples of the use of ion mobility spectrometry can also be found in the literature [8,9,10]. The topic of various techniques for detecting explosives is extensively described in several excellent reviews [11,12,13].

The analytical techniques mentioned above enable the operation of many devices capable of detecting low concentrations of explosive substances, often at ppm levels and below. Although this type of apparatus provides very precise results and is extremely sensitive, it is often very expensive and requires highly qualified personnel to operate it. For this reason, in certain situations it is necessary to use portable and easy-to-use devices for detecting explosives. Such devices include various chemical sensors, which are generally less sensitive and selective than the aforementioned analytical methods, but are ideal for use in a variety of environmental conditions, such as crime scenes. Chemical sensors yield fast results and are relatively inexpensive to produce, making them of great interest to many research groups. Reviews on the design of chemical sensors for the detection of explosives are available in the literature [14,15], in addition to plenty of papers describing various sensors and detection methods developed for many common explosives [16,17,18,19,20,21,22,23,24,25,26].

An interesting group of sensors are paper-based sensors, also known as micro Paper Analytical Devices (µPADs). Although µPADs are single-use, which may make it debatable whether they can be called sensors, they are a very attractive alternative to many devices since they do not require any power supply. Furthermore, detection is usually based on color evaluation, which can be performed visually [27,28,29,30,31]. Color reactions are among the oldest and simplest methods in analytical chemistry. They exploit the principle that a specific compound or group of compounds, when treated with an appropriate reagent, triggers a chemical reaction that leads to the formation of a colored product characteristic of that compound or group of compounds. Excellent examples of color reactions can be found in the books by F.Feigl [32], by E.Jungreis [33], as well as in a review by M.T. Doménech-Carbó [34]. The spot tests, as historical predecessors of lab on paper sensors, were performed on a white porcelain plate or with the application of filter papers. Among many applications, paper-based sensors have also been successfully applied to explosive material detection [35,36,37].

Color reactions are widely used in explosive analysis because they are easy to perform and do not require sophisticated equipment. They allow rapid and on-site detection of explosives and are utilized for preliminary laboratory testing of materials suspected of being explosives. However, the main limitation of using color reactions in explosive analysis is their inherently low specificity. The identification of an explosive cannot be unequivocally confirmed based on a color reaction alone, as other non-explosive compounds can also produce the same color under identical experimental conditions. Moreover, adapting many well-known chemical reactions, used for the determination of explosive materials, for paper-based sensors is often challenging. Specifically, the application of certain necessary reagents that evaporate quickly or are unstable presents significant practical problems.

Fast and simple detection of explosives is a critical issue that necessitates an individualized approach for each substance being tested. Discussions about the detection of homemade explosives (HMEs) usually focus on the detection of the two most common compounds in this group: triacetone triperoxide (TATP), and hexamethylene triperoxide diamine (HMTD). Triacetone triperoxide was synthesized by Wolffenstein [38], and hexamethylene triperoxide diamine was synthesized by Legler at the end of the XIX century [39]. Neither of these materials are typically employed by military or professional personnel; however, their detonation can cause substantial damage compared to well-known materials such as trinitrotoluene. Crucially, these materials are extremely sensitive to friction, shock, heat, and electrostatic discharge, which makes them highly problematic from a handling and application standpoint. Consequently, their transportation, storage, and handling must be performed with utmost care.

From a chemical point of view, peroxide structures like TATP and HMTD lack nitro groups or aromatic rings. Thus, the methods developed for the determination of nitrogenous groups in well-known explosive materials, such as 2,4,6-trinitrotoluene (TNT), 2,4-dinitrotoluene (2,4-DNT), 2,6-dinitrotoluene (2,6-DNT), hexahydro-1,3,5-trinitro-1,3,5-triazine (RDX), and 1,3,5,7-tetranitro-1,3,5,7-tetrazacyclooctane (HMX) cannot be directly applied or easily modified. This highlights the critical need for a fast and reliable method for HME detection. The field has been comprehensively reviewed in several papers [40,41,42]. Typically, HME detection methods rely on the utilization of concentrated acid to decompose the HME into hydrogen peroxide, and then, a product of such a reaction can be detected [43,44,45,46,47]. An alternative approach involves the photodecomposition of TATP or HMTD using UV light (254 nm), followed by the detection of the degradation product, H_2_O_2_ [48,49,50].

Non-standard methods found in the literature include: the application of modified glassy carbon electrodes imprinted for molecular recognition [51], the application of KBr and heat, followed by chronoamperometric techniques [52], and the application of nanozymes [53]. Lin and co-workers [54] used a solid acid catalyst to pretreat a gaseous TATP, followed by a colorimetric sensor array of redox-sensitive dyes.

This paper presents a fast colorimetric method for the visual detection of HMTD based on the application of µPAD. The developed colorimetric reaction is based on the use of a potassium iodide and citric acid solution. The applicability of the novel colorimetric reaction performed on µPADs was compared with commercially available and homemade iodometric tests, which have previously been reported to be useful for detecting peroxides [55,56].

## 2. Materials and Methods

HMTD and TATP were synthesized by the Military Institute of Armament Technology (Zielonka, Poland). To facilitate measurements using paper-based sensors, appropriate solutions of the explosive materials were prepared. Working with solutions of explosive materials is significantly safer than working with solid-state samples. A mixture of acetone/water (50:50%) was used to prepare the solutions. The stock solution was prepared at a concentration of 1 mg/mL. This stock solution was then diluted to obtain the required working concentrations.

Potassium iodide (KI) and starch were purchased from Warchem (Zakręt, Poland) and were used without any further purification. Solutions of KI and starch were prepared in deionized water at concentrations of 10% and 10%, respectively. Hydrogen peroxide (30%) and citric acid were purchased from Merck (Poznań, Poland). Whatman chromatography paper No. 1 was purchased from GE (Tonglu, China) and used for the fabrication of paper-based sensors. Potassium iodide starch paper MN 816 N was purchased from Macherey-Nagel (Düren, Germany).

## 3. Results

### 3.1. Fabrication of Sensors

**Figure 1:**
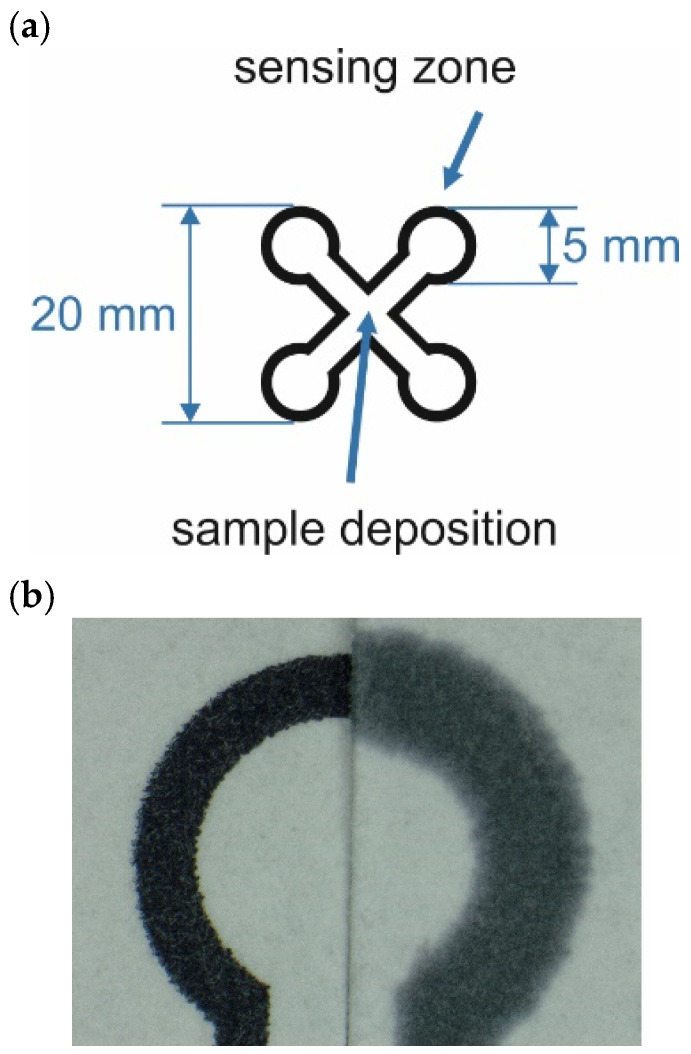
(**a**) Architecture of the paper-based sensor and (**b**) photograph of the sensing zone before (left) and after heating (right).

The chemical sensors were fabricated using Whatman chromatography paper No. 1, which was patterned with a wax barrier. The sensor architecture was designed using CorelDraw Graphics Suite 2019 software. Figure 1 presents the architecture of the μPAD sensor.

The sensor incorporates four circular sensing zones where a chemical indicator can be deposited. The sample under test was dispensed onto the central point of the sensor, and then capillary forces delivered the sample to the sensing zones. These four sensing zones were designed to enable future multianalyte detection and/or allow for calculating an average colorimetric response for the same sample.

A precise wax pattern was deposited using a Colorqube 8700 Xerox wax printer (Xerox, Warsaw, Poland). Subsequently, the chromatography paper was heated to 170 °C for 2 min in a laboratory oven. This process allows the wax to penetrate the paper support, thus creating a robust hydrophobic barrier. Initially, the sensing zone was designed with a diameter of 5 mm. Following the heating process, the inner diameter decreased to 3.5 mm (see Figure 1b). This shrinkage occurs because the molten wax penetrates the paper while simultaneously spreading laterally on its surface. It was experimentally determined that the volume of the indicator dispensed onto the sensing zone was 0.5 μL, whereas the total volume of the sample under test was 10 μL. A precise micropipette (Mettler Toledo, Warsaw, Poland) was used for dispensing the liquids.

Literature reports indicate that the orientation of paper fibers is often non-uniform [57,58]. This non-uniformity is inherent to the paper fabrication process. Thus, assessing the fiber orientation for each new sheet of paper, taken directly from the packaging, is impractical. A specialized experimental procedure can be applied, based on measuring the diameter of the spot created by a color indicator. The difference in spot diameter along the X- and Y-axes can reveal the fiber orientation. However, applying this procedure to every single sheet of paper would render the entire preparation process prohibitively time-consuming. Considering these limitations and mitigating variations in capillary flow, the sensor architecture was designed such that the microfluidic (capillary) channels were oriented at a 45° angle relative to the paper’s edge. We hypothesized that capillary flow would be uniform in every direction, assuming the fiber orientation would be similar across all microfluidic channels. However, the authors did not perform further experiments to confirm this phenomenon, as the focus of this paper was the colorimetric detection of peroxides.

### 3.2. Colorimetric Determination of Peroxides

#### 3.2.1. The Usage of Commercial Starch-Iodometric Test

**Figure 2:**
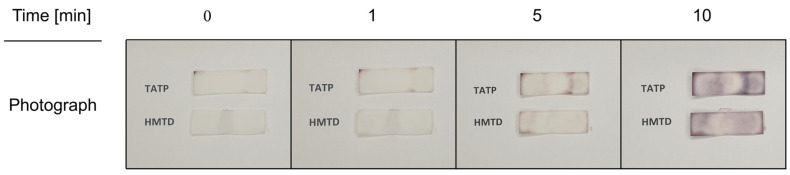
Photographs of commercially available iodide/starch paper treated with homemade explosives. The concentrations of TATP and HMTD were 1 mg/mL.

Peroxides can be determined colorimetrically using a well-established reaction. The concentration of peroxides directly influences the intensity of the color developed. Various reagents can be employed for peroxide determination. One of the most popular is potassium iodide (KI), which reacts with peroxides to produce iodine. This liberated iodine can then react with chromogenic reagents (such as starch) to form colored complexes. However, the colorimetric determination of peroxides is subject to certain limitations. Firstly, the method may lack specificity toward a particular type of peroxide, as certain reagents can react with other compounds present in the sample. Furthermore, interference from other substances in the sample can also compromise the accuracy of the measurement. Therefore, it is crucial to select appropriate reagents and carefully consider potential interferences. The well-established colorimetric determination of hydrogen peroxide, utilized in analytical chemistry for many years, is based on the starch-iodide reaction [59]. The explosive materials investigated in this study belong to the peroxide group; however, they are less potent oxidants than H_2_O_2_. Initially, we sought to determine whether commercially available starch-iodide papers could be employed for the colorimetric detection of explosive peroxides, specifically TATP and HMTD. Figure 2 presents photographs of starch paper strips treated with solutions of TATP and HMTD.

A solution (70 µL) of TATP or HMTD was dispensed onto the centre of the paper strip. The initial observation (as shown in the first image) was recorded immediately after solution contact. Initially, the paper strips exhibited no visible color change when treated with HME (Home Made Materials). Only a minor change was noted at the edges of the paper. The paper strips were left to react with the HME under ambient laboratory conditions. After a few minutes, a slightly non-uniform color change became apparent. The color change became distinctly more visible after 10 min. Thus, while the commercial starch paper exhibited some response to HME, the observed reaction time is quite long from a practical standpoint, and the response itself is non-selective. Specifically, it is impossible to distinguish between the types of HME used (TATP vs. HMTD).

The experiments confirmed that a well-established reaction based on potassium iodide can be successfully applied to HME testing. It is evident that the specific details concerning the starch paper composition are proprietary and cannot be disclosed by the manufacturer. Based on publicly available information, such as the Safety Data Sheet [60], it can be deduced that the paper substrate consists of cellulose, indicator dyes (i.e., KI and starch), surfactants, polymers, and buffers. The latter compounds (surfactants, polymers, and buffers) are likely incorporated to ensure the paper’s long shelf life and maintain the optimal reaction pH.

An intriguing issue arises regarding the relatively long induction time required for the commercial papers to exhibit a color change. The resulting color, expected from the well-established reaction, is dark purple, yet it should theoretically manifest much faster upon contact with peroxides. Therefore, it must be investigated whether this delay is attributable to the extended time necessary for potassium iodide to react with the individual peroxides, or if the observed color is the result of a secondary, more complex reaction.

#### 3.2.2. The Usage of Homemade Starch-Iodometric Test

An interesting study details the application of starch-iodide-gelatin systems for hydrogen peroxide and glucose determination [55]. The authors developed a microfluidic paper-based sensor for hydrogen peroxide and glucose determination. They utilized a well-known starch-iodide composition, and moreover, the sensing layer was covered with gelatine to protect iodine from oxidation. Inspired by the methodology described in this work, we subsequently prepared a lab-on-paper sensor for the detection of explosive peroxides.

**Figure 3:**
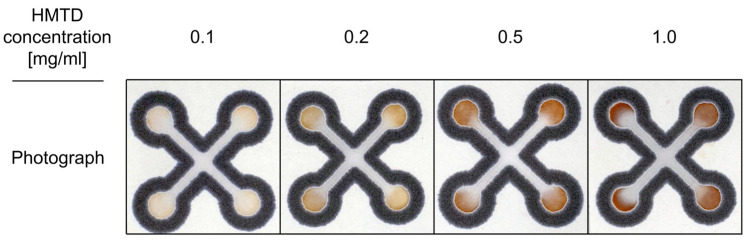
Photographs of the starch-iodide sensor treated with HMTD at various concentrations.

Aqueous solutions of KI (10%) and starch (10%) were prepared, mixed (1:1 *v*/*v*), and vortexed. A volume of 0.5 µL of the resulting indicator solution was dispensed onto each sensing zone. A precise micropipette was utilized for the deposition of the solution onto the paper-based sensors. The sensors were then allowed to dry in the laboratory for 5 min. Following sensor preparation, we investigated the influence of various concentrations of HMTD and TATP on the colorimetric response. Specifically, no color change was observed when testing TATP. Representative photographs of sensors treated with HMTD are presented in Figure 3.

The color of the sensing zones changed from neutral (colorless) to brown—red. The total response time was approximately 1 min, which is considered acceptable from a practical standpoint. The limit of detection (LOD) for this method is as low as 0.2 mg/mL.

We subsequently investigated the influence of other explosive materials on the designed sensors to assess selectivity. Crucially, no color change was observed when testing solutions of TNT, 2,4-DNT, 2,6-DNT, RDX, HMX, or PETN.

#### 3.2.3. Colorimetric Determination of Peroxides Using Novel Colorimetric Reaction

**Figure 4:**
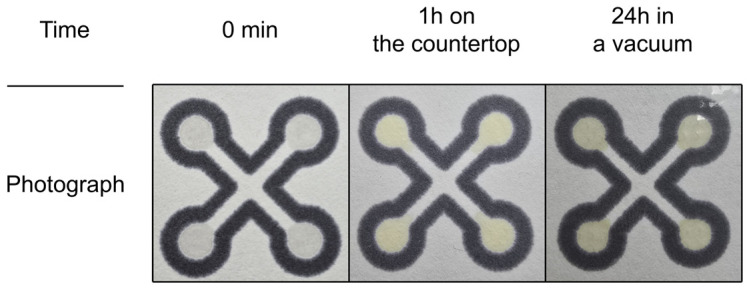
Photographs of the sensors stored under different conditions.

The reaction discussed in this section was discovered when we faced the problem of how to use the methods described in the literature for determining HME using paper sensors. The primary assumption was the utilisation of an acid which could decompose HME into H_2_O_2_, and then a chemical indicator deposited on paper sensor would detect hydrogen peroxide. Such an acid should be deposited on the paper surface, dry, and then could be used to perform the reaction. It was impossible to utilise any strong acid as it was reported in the Refs. [43,44,45,46,47]. Typical strong acids would evaporate or destroy the paper. Thus, we decided to use citric acid. The indicator solution utilized for colorimetric analyses, hereafter referred to as “Mix,” consists of citric acid and 10% potassium iodide mixed in a 1:1 m:v ratio (this optimal ratio was determined experimentally). Experiments demonstrated that optimal results were achieved by applying 0.5 µL of Mix twice (1 µL in total). Immediately after preparation, Mix is colorless, but it gradually turns slightly yellow over time, as shown in Figure 4.

Prolonged exposure of the sensor to ambient laboratory air results in the decomposition of potassium iodide, causing the detection zones to turn yellow. For this reason, following preparation, the sensors are stored under vacuum, a measure which significantly delays reagent degradation and greatly extends their long-term stability.

**Figure 5:**
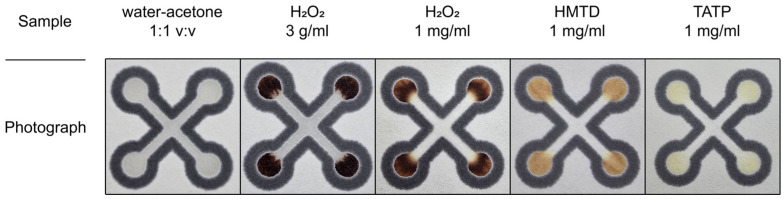
Photographs of the sensor treated with various solutions.

Following sensor preparation, we assessed the influence of various control solutions on the colorimetric response. Specifically, no color change was observed upon exposure to the pure acetone/water solution (the solvent used to prepare the explosive samples). Representative photographs of the tested sensors are presented in Figure 5.

The total response time was less than 1 min. The areas where H_2_O_2_ is applied initially exhibit an orange-brown coloration, which rapidly darkens to black or dark brown, depending on the concentration. Interestingly, at a concentration of 3 g/mL H_2_O_2_ (which corresponds to the concentration of peroxide in hydrogen peroxide), the sensing zone begins to fade after a few minutes until it becomes completely colorless. In contrast, the color obtained on the sensors is stable over time for the HMTD samples. For the HMTD samples, the detection zones turn orange-brown, whereas for the TATP samples, they exhibit a pale-yellow coloration.

**Figure 6:**
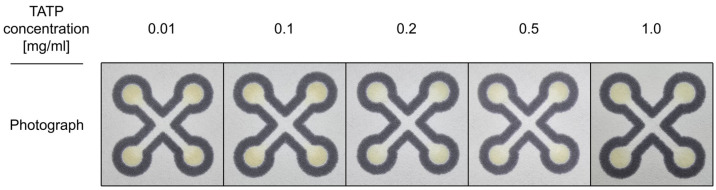
Photographs of the sensor treated with TATP at various concentrations.

**Figure 7:**
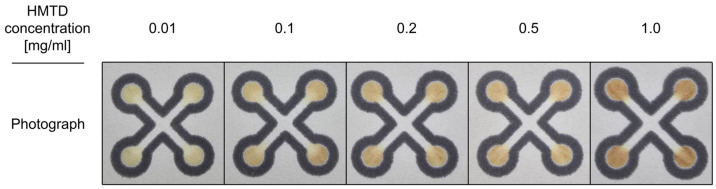
Photographs of the sensor treated with HMTD at various concentrations.

Subsequently, we investigated the influence of various concentrations of HMTD and TATP on the colorimetric response. Representative photographs of sensors treated with TATP and HMTD are presented in Figure 6 and Figure 7, respectively.

Regardless of the TATP concentration, the color change was consistent, ranging from neutral to light yellow. The color observed in the TATP detection zones is distinguishable from the control (pure acetone/water mixture), yet this difference is subtle and comparable to the slight discoloration exhibited by a dry sensor stored at ambient conditions for one hour. Sensors prepared with Mix are suitable for detecting even small concentrations of TATP immediately after preparation, but due to slight yellowing over time, they lose their usefulness during storage. An interesting observation concerns the long-term stability: both the dry sensor and the sensor treated with TATP show an initial increase in yellowing intensity over time. Crucially, after a few days, the unreacted dry sensor completely fades (discolors), while the sensor treated with the HMTD sample retains its color and remains stable.

Comparing the results obtained with the publication [61], which also tested sensors for HMTD and TATP, it can be concluded that our solution is highly selective. TATP causes a slight yellow discoloration of the detection zone, the intensity of which does not depend on the concentration of TATP. When testing HMTD samples, a clear change in the color of the detection zone to orangebrown can be observed. The sensor’s response time is very short—less than 1 min, which is significantly shorter than in [61]. The authors of this publication state that their sensor provides reliable results after 15 min. In our solution, the sensor response time also includes the time needed for the sample to reach the detection zone due to capillary flow from the center point of the sensor. In Ref. [61], the test sample was applied directly to the detection zone and there was virtually no delay caused by capillary forces. Table 1 presents a comparison of the performance of both sensors, as well as a comparison with the peroxide detection solution patented by L. Yushu’s team [62].

**Figure 8:**
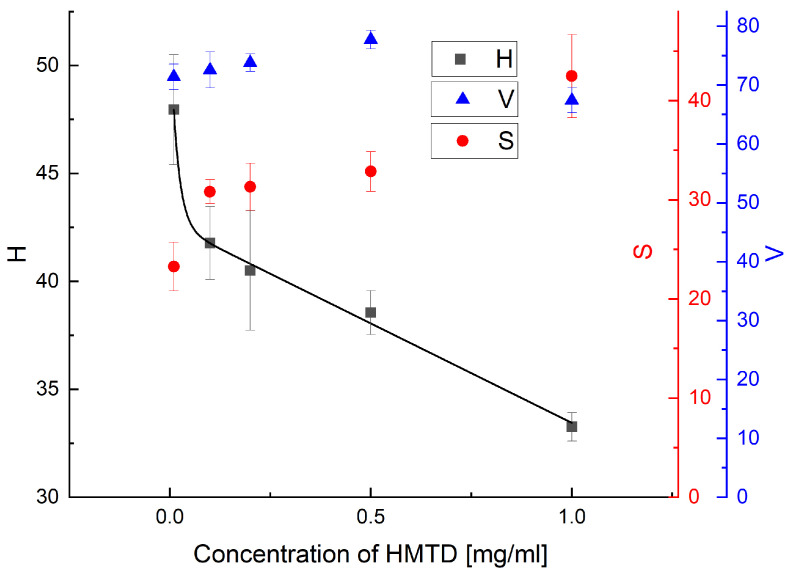
HSV analysis of the photographs from Figure 7. For each sensor, all four sensing zones were analyzed.

All acquired images were examined utilizing the Trigit software. Trigit is an interactive web application available online [63]. The software was developed at The University of New South Wales (UNSW Sydney, Australia) [64]. It offers automatic pixel selection for examination, which is particularly advantageous when non-uniform color distribution occurs. Trigit also offers the option of selecting a specific, desired area and standardizing the colors within that area. The option of selecting a specific area—the entire detection zone—was chosen for colour assessment in this work. There are four color spaces available for selection: RGB, CMYK, CIElab, and HSV. The RGB color model is the most popular color model used in digital image processing, commonly utilized by digital photography. A color image is composed of three channels, i.e., red, green, and blue. All other colors are formed only by the proportional ratio of these three colors. In the HSV model, each image pixel is described by three channels: hue, saturation, and value. This model does not directly use primary colors. It uses colors in the way people perceive them. Crucially, the color information is determined by only one value, i.e., hue, and that is why we decided to present HSV analysis. The results obtained from the Trigit software measurements (5 replicates for each concentration) are presented in Figure 8.

A color change was observed across the detection zones of all sensors treated with the HMTD solution. The highest concentrations of this explosive material resulted in orange-brown coloration. The remaining zones were characterized by an orange-brown hue with varying saturation levels. Importantly, no linear relationship between the color component (Hue) and HMTD concentration was observed within the tested range. In the case of our sensor, the LOD obtained is significantly better compared to the work [61]. This publication gives an LOD of 1 µg in a 10 µL sample volume, which can be converted to 0.1 mg/mL. In our case, we can determine the concentration at 0.01 mg/mL. Nevertheless, the developed sensor represents a highly suitable and inexpensive solution for the qualitative analysis and detection of this specific explosive, effectively distinguishing it from hydrogen peroxide.

## 4. Discussion

The detection of homemade explosives (HMEs) is paramount from a public security standpoint. While numerous specialized devices can detect these hazardous substances at concentrations in the parts-per-million (ppm) range, such equipment is often expensive, bulky, heavy, and requires a qualified operator. Consequently, there is an urgent need for cheaper and simpler analytical alternatives.

The use of peroxide activity is a very simple and effective method for colorimetric detection of analytes, while paper substrates are considered one of the most promising materials to produce inexpensive and easy-to-use sensors [65,66]. Therefore, we designed and successfully tested a paper-based chemical sensor capable of colorimetrically detecting hexamethylene triperoxide diamine (HMTD). The detection mechanism relies on the color change of “Mix” (a proprietary solution of potassium iodide and citric acid) upon contact with peroxides. Our sensor is highly light-weight, portable, and inexpensive, offering naked-eye detection based on visible color formation.

The developed sensor enables HMTD detection at a concentration of 0.01 mg/mL with a response time of less than one minute. These features make the sensor an excellent tool for rapid, preliminary detection of potential hazards in field settings where complex equipment access is limited. A major advantage of the developed paper sensor is its ability to differentiate between hydrogen peroxide and explosive peroxide samples, as distinct colors are generated in the detection zones for each.

## Figures and Tables

**Table 1:** Comparison of approaches using potassium iodide for the detection of explosive peroxides.

	This Work	[61]	[62]
detected peroxide	HMTD	HMTD, TATP	TATP
sensor used	wax printed paper-based sensor	wax printed paper-based sensor	unspecified container for liquid reagents
response time	1 min	at least 5 min up to 15 min	no data
detection method	‘naked eye’	image analysis (developed iOS application)	‘naked eye’
reagents	-potassium iodide -citric acid	-potassium iodide -hydrochloride acid	-potassium iodide -sulfuric acid
LOD	HMTD: 0.01 mg/mL	tested concentrations: HMTD: 2.1 mg/mL TATP: 2.2 mg/mL	TATP: 0.1 mg/mL
selectivity	Sensor insensitive to TATP, TNT, DNT, RDX, HMX and PA	Different color for TATP, sensor insensitive to NB, 4A2NP and PA	no data

## Data Availability

The original contributions presented in this study are included in the article. Further inquiries can be directed to the corresponding authors.
